# A single-band ratiometric luminescent thermometer based on tetrafluorides operating entirely in the infrared region†

**DOI:** 10.1039/d1na00727k

**Published:** 2021-11-12

**Authors:** K. Trejgis, K. Ledwa, A. Bednarkiewicz, L. Marciniak

**Affiliations:** Institute of Low Temperature and Structure Research, Polish Academy of Sciences Okolna 2 50-422 Wroclaw Poland k.trejgis@intibs.pl l.marciniak@intibs.pl

## Abstract

Luminescence thermometry is a remote temperature measurement technique that relies on thermally induced changes in spectroscopic properties. Because of its great application potential, even under very demanding conditions where other techniques fail, it has attracted the attention of many researchers in recent years. Unfortunately, most of the existing luminescence thermometers are fraught with large inaccuracies and thus are not reliable enough to be applied in real and demanding applications. However, there is one of the most recent and very promising but insufficiently studied approaches to luminescence thermometry quantification – single-band ratiometric luminescence thermometry. It is based on the analysis of the luminescence intensity ratio of a single emission band being photoexcited in two ways, *i.e.* by ground (GSA) and excited (ESA) state absorption. It is characterized by high relative sensitivity to temperature changes as well as high measurement precision. However, because ESA-excited luminescence intensity can depend on the type and concentration of dopant ions or the properties of the host material, further more-detailed studies must be conducted to understand the impact of numerous photophysical processes on the relative sensitivity, temperature resolution and useful temperature range of SBR LTs. In this work, the effect of interionic interactions occurring through cross-relaxation on the thermometric properties of single-band ratiometric luminescent thermometers in NaYF_4_:Nd^3+^ and NaGdF_4_:Nd^3+^ was investigated and discussed. In contrast to the disadvantageous concentration quenching phenomenon that is typically observed at an increased content of dopants, the beneficial role of cross-relaxation in the enhancement of the signal-to-noise ratio of the ESA-excited luminescence at high temperatures was demonstrated. The maximum relative temperature sensitivity reached was equal to *S*_R_ = 16.9% K^−1^ at 223 K for NaYF_4_:50%Nd^3+^ nanocrystals and its value remained above 1% K^−1^ throughout the whole analyzed temperature range from 223 K to 473 K.

## Abstract 2

Luminescence thermometry is a remote temperature measurement technique that relies on thermally induced changes in spectroscopic properties.

## Graphical abstract

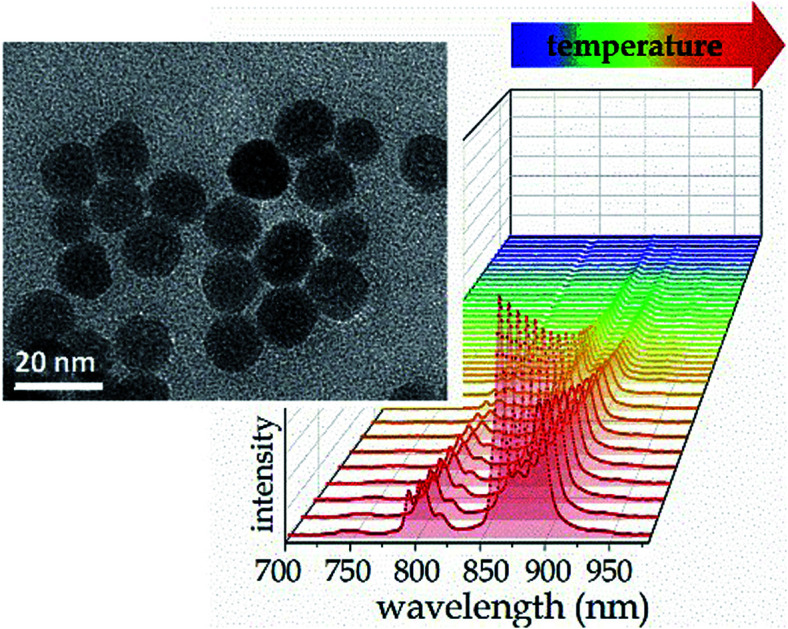

## Introduction

Temperature is one of the most frequently measured and most essential quantities to understanding physical, chemical or biological phenomena; therefore high detection precision is one of the prominent requirements imposed on metrology. Recently, a lot of interest has been directed towards luminescence thermometry (LT), which enables temperature measurement based on changes in the spectroscopic properties of the phosphor, which plays the role of a thermometer.^1–3^ A particular advantage of this technique is that it can be measured in a remote manner, allowing the luminescent thermometer to be used even under very demanding conditions such as rotating components of engines during the operating time, high-temperature smelting furnaces, microelectronic systems and even biological applications.^2,4–6^ In order to intentionally design a highly temperature-sensitive luminescent thermometer, the phosphor properties and performance as well as the temperature readout methodology must be further optimized.

Until recently, 6 classes of thermometers were distinguished, among which were mainly those in which the temperature-dependent parameter was based on the luminescence intensity ratio (LIR) of two emission bands of phosphors being utilized as a thermometric parameter.^7–14^ However, the main disadvantage of this method is associated with the need to spectrally separate emission signals involved in the ratiometric readout. This may not be an easy task, especially when the two emission bands are spectrally overlapping or are in very close vicinity. For single spot temperature measurement high spectral resolution is required, which is feasible to be acquired with modern CCD spectrometers. However, when temperature 2D imaging is the target application, hyperspectral tunable filters or switching between narrowband dichroic spectral filters is required, which makes the readout technically complicated or costly, and it further hampers the precision and reliability of temperature imaging. What is more, due to the dispersive dependence of the extinction coefficient of the medium in which the thermometer is embedded, each band can be modulated differently (by absorption or scattering in the sample) affecting the luminescence ratio of the thermometer and thus the precision of the measurement. These issues and observations became the inspiration to develop a new approach – *i.e.* the single-band ratiometric (SBR) technique in which a single emission band is engaged for temperature determination, which overcomes the aforementioned limitations.^15–17^ To ensure ratiometricity, *i.e.*, the presence of two signals to be compared against each other, while maintaining the single band character, two excitation wavelengths are used in SBR LT, *i.e.*, one pumping beam being resonant with ground state absorption (GSA) and the other one matching excited state absorption (ESA). As the temperature increases, the intensity of GSA-excited luminescence decreases due to several processes called luminescence thermal quenching among which the multi-phonon relaxation is the dominant one in the lanthanide ions (Ln^3+^). On the other hand, ESA-excited luminescence is observed only when the population of the excited level is sufficient for the absorption process from that level to take place, whereby the most effective channel for its loading, according to the Boltzmann distribution, is the thermal energy supplied to the system. Thus, as the temperature increases, the population of the excited level also increases resulting in a strong enhancement of ESA-excited luminescence intensity. This opposite characteristic of luminescence intensity changes, which is dependent on the excitation wavelength, demonstrates the potential to develop a novel, efficient and highly sensitive tool to quantify or image temperature.

In the ESA process, the population of the level from which absorption takes place and thus also the ESA-excited emission intensity, depends on various factors such as the concentration of optically active ions, surface effects, host phonon energy, crystal structure and others.^15–17^ Moreover, only selected dopant ions enable the ESA process to occur and one of the flagship examples of such ions are Nd^3+^ ions, which are characterized by a *ca.* 2000 cm^−1^ energy difference between the ground ^4^I_9/2_ level and the first excited ^4^I_11/2_ level from which ESA occurs. Such an energy gap can be overcome thermally, which enables the population density in the first excited ^4^I_11/2_ level to be increased and trapped there to further increase the signal-to-noise ratio of the ^4^F_3/2_ → ^4^I_9/2_ emission upon excitation with 1060 nm. An additional advantage of Nd^3+^ ions owes to the possibility to promote the (^4^F_3/2_; ^4^I_9/2_) → (^4^I_15/2_; ^4^I_15/2_) cross-relaxation (CR) phenomenon, which, as a result of the subsequent nonradiative relaxation to lower ^4^I_*J*_ states, can be a very efficient source of excited level population. This phenomenon is very beneficial for the intensity enhancement of ESA-induced luminescence. Moreover, a characteristic feature of Nd^3+^ ions is the possibility of excitation and detection in the near-infrared (NIR) region,^18,19^ which is advantageous for the applications in the biomedical field. This is because NIR radiation falls in the biological optical window within which the scattering and absorption of light on the body tissues are reduced.^20^ Due to all the mentioned factors, Nd^3+^ ions are considered in this work.

An important aspect of luminescence thermometry is the capability to rationally select, design and synthesize efficient, photochemically and thermally stable host materials. The hosts characterized by high phonon energies are considered unfavorable for luminescence thermometry applications; therefore, low phonon energy materials, such as fluorides, which also satisfy the photochemical and thermal stability conditions, seem to be most suitable. Taking this into account, two fluoride hosts NaYF_4_ and NaGdF_4_ doped with Nd^3+^ ions are analyzed in this work.^21^ Additionally, because the cross-relaxation phenomenon is at least an order of magnitude stronger in the hexagonal phase than in the cubic counterpart,^22,23^ it is the hexagonal phase that is analyzed in this work.

In order to understand the impact of interionic interactions on the spectroscopic and thermometric properties of the single-band ratiometric thermometers based on nanocrystalline fluoride NaYF_4_ and NaGdF_4_ hosts doped with Nd^3+^ ions, seven dopant ion concentrations ranging from 0.1% to 50% are analyzed in this work. This research contributes to a better understanding of the factors affecting the ESA process, which is required to move towards the development of an optimal, highly sensitive luminescence thermometer.

## Experimental

### Materials preparation

The materials were synthesized by the thermal decomposition method in boiling oleic acid and octadecene acting as solvents.^24^

Neodymium(iii) acetate hydrate (99.9%), yttrium(iii) acetate hydrate(99.9%), ammonium fluoride (99.99%), acetic acid (99%), pure oleic acid and 1-octadecene (90%) were purchased from Sigma Aldrich. Sodium hydroxide (99.8%), ethanol (96% pure p.a.), methanol, *n*-hexane and chloroform were purchased from POCH S.A. (Poland). All of the chemical reagents were used as received without future purification.

The synthesis was performed for 1.50 mmol of final product. Depending on the dopant ion concentration, appropriate amounts of neodymium acetate and either yttrium acetate or gadolinium acetate depending on the matrix synthesized (NaYF_4_ and NaGdF_4_) were placed in a 250 mL three-neck round-bottom flask along with 22.5 mL of octadecene and 9 mL of oleic acid. The solution was then magnetically stirred and heated slowly to 140 °C under vacuum conditions and further stirred at this temperature for 30 min to form Y(oleate)_3_ and to remove residual water and oxygen. At the same time, 0.22222 g (6 mmol) of ammonium fluoride and 0.14999 g (3.75 mmol) of sodium hydroxide were weighed into another vessel and 10 mL of methanol was added and magnetically stirred together. In the next step, the temperature of oleates was reduced to 50 °C and the atmosphere was changed from vacuum to a gentle flow of nitrogen. When the temperature of the solution reached 50 °C, a methanol solution of NaOH and NH_4_F was added quickly to the flask through the side neck and stirred under these conditions for 30 minutes. After this time, the temperature was increased to 85 °C and the conditions were changed to vacuum to completely evaporate the methanol from the reaction mixture. After the methanol evaporation, the reaction temperature was increased to 300 °C as quickly as possible and maintained at this temperature for 60 minutes under a nitrogen flow. The final transparent solution was then cooled to room temperature. The NPs were precipitated by addition of ethanol and isolated by centrifugation at 10 000 rpm for 10 min. For purification, the resulting pellet was dispersed in a minimal amount of *n*-hexane and again precipitated with excess ethanol. The UCNPs were isolated by centrifugation at 14 000 rpm for 10 min. The final product stabilized with OA ligands was dispersed in 3.75 cm^3^ of chloroform (CHCl_3_).

Samples for spectroscopic measurements were prepared by adding dropwise 0.75 μL onto a glass slide and evaporating the chloroform.

### Characterization

Powder diffraction data were obtained using a PANalytical X'Pert Pro diffractometer equipped with an Anton Paar TCU 1000 N Temperature Control Unit using Ni-filtered Cu Kα radiation (*V* = 40 kV and *I* = 30 mA). Transmission electron microscope (TEM) images were recorded with a Philips CM-20 SuperTwin transmission electron microscope, operating at 160 kV. A drop of the suspension was put on a copper microscope grid covered with carbon. Before the measurement, the sample was dried and purified in a H_2_/O_2_ plasma cleaner for 1 min. The hydrodynamic size of nanoparticles was determined by dynamic light scattering (DLS) conducted by using a Malvern ZetaSizer at room temperature in a quartz cuvette using chloroform as a dispersant. The excitation spectra and luminescence decay profiles were obtained using an FLS1000 Fluorescence Spectrometer from Edinburgh Instruments equipped with a 450 W xenon lamp and μFlash lamp as an excitation sources and R5509-72 photomultiplier tube from Hamamatsu in a nitrogen-flow cooled housing as a detector. To carry out the temperature measurement, the temperature of the sample was controlled using a THMS 600 heating–cooling stage from Linkam (0.1 °C temperature stability and 0.1 °C set point resolution). The emission spectra were recorded using 808 nm and 1060 nm excitation lines from laser diodes (LDs) and a Silver-Nova Super Range TEC Spectrometer from Stellarnet (1 nm spectral resolution) as a detector.

## Results and discussion

**Fig. 1:**
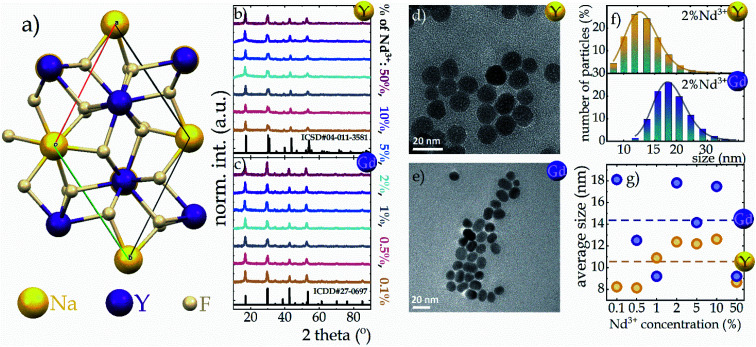
Visualization of Na^+^ and Y^3+^ sites in the hexagonal NaYF_4_ structure (a); Bragg reflections of NaYF_4_ (b) and NaGdF_4_ (c) nanocrystals with different Nd^3+^ concentrations; the representative TEM images of NaYF_4_:2%Nd^3+^ (d) and NaGdF_4_:2%Nd^3+^ (e) nanocrystals; the hydrodynamic size distribution of representative NaYF_4_:2%Nd^3+^ (f, top) and NaGdF_4_:2%Nd^3+^ (f, bottom) determined from DLS measurement; the dopant ion concentration impact on the average hydrodynamic size of both nanocrystalline hosts (g).

As is well known, sodium tetrafluorides crystalize into either a cubic (α) or a hexagonal (β) crystallographic phase. However, due to the well-known fact that much more efficient luminescence is observed in the hexagonal phase than in its cubic counterpart,^22,23^ the β phase of NaYF_4_ and NaGdF_4_ was investigated in this work. A characteristic feature of β-NaYF_4_ is an ordered arrangement of F^−^ ions in the vicinity of two cationic sites, one of which is completely occupied by Y^3+^ ions and the other one only partially by Y^3+^ ions and partially by Na^+^ ions^25–28^ (Fig. 1a). Identical behavior occurs for the NaGdF_4_ host (Gd^3+^ instead of Y^3+^ ions). Moreover both analyzed hosts adopt the same *P*6̄ symmetry group.^25^ However the change from the smaller Y^3+^ ion (ionic radius of 0.900 Å ^29^) to the larger Gd^3+^ ion (ionic radius of 0.938 Å ^29^) increases the unit cell parameters from *a* = *b* = 5.96 Å and *c* = 3.53 Å of NaYF_4_ to the larger *a* = *b* = 6.02 Å and *c* = 3.60 Å of NaGdF_4_. The enlargement of the unit cell volume (see Fig. S6†) is evidenced by a shift of the Bragg reflections characteristic for the NaGdF_4_ host toward lower angles relative to those for NaYF_4_ ^30^ (Fig. 1b, c and S1b†). Moreover, at high levels of Nd^3+^ ion doping (∼50%) for both hosts, a subtle shift of the Bragg reflection maxima towards lower angles is noted, whereby for the NaYF_4_:50%Nd^3+^ host this shift is more pronounced than for NaGdF_4_:50%Nd^3+^ (Fig. S1†). The reason for this is probably also related to the enlargement of the unit cell volume due to the substitution of Y^3+^ or Gd^3+^ ions with Nd^3+^ ions, which have a larger ionic radius of 0.983 Å, and because of the larger size difference between Y^3+^ and Nd^3+^ ions than between a pair of Gd^3+^ and Nd^3+^ ions, this shift is more prominent in the NaYF_4_ host. In addition, the Bragg reflections reveal broadening relative to the standard pattern, which is indicative of the small size of the nanocrystallites. This was confirmed by the TEM images presented in Fig. 1d and e (for NaYF_4_ and NaGdF_4_, respectively) and also by the hydrodynamic size distribution determined from DLS measurements seen in Fig. 1f (for other concentrations see Fig. S2†). Morphological analysis shows that non-aggregated, homogeneous nanocrystals with a narrow size distribution were obtained. In the case of the NaYF_4_ host, a spherical shape with an average size of about 10–12 nm is observed (Fig. 1g), while, as expected, the NaGdF_4_ nanoparticles assumed a slightly elongated shape with a slightly larger size *i.e.* 18 nm for NaGdF_4_:2%Nd^3+^ and about 14 nm on average for all concentrations analyzed (Fig. 1g). As previous studies have shown, the different shapes of these materials may be due to differences in the atomic number and also the ionic radius of RE^3+^ ions.^30,31^

**Fig. 2:**
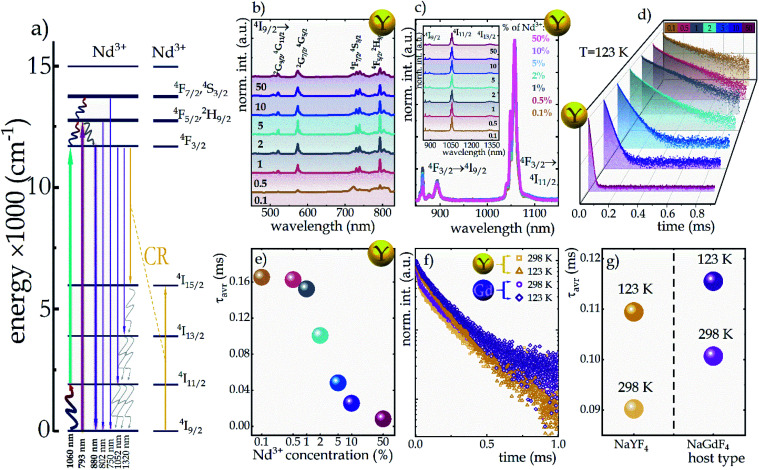
Simplified energy diagram of Nd^3+^ ions (a); Nd^3+^ ion concentration impact on excitation spectra monitored at 1058 nm in NaYF_4_:Nd^3+^ nanocrystals (b), the comparison of the emission spectra upon 793 nm excitation for NaYF_4_:Nd^3+^ (c), the luminescence decay profiles of NaYF_4_:Nd^3+^ of the excited ^4^F_3/2_ state measured at 123 K (d) and average decay time of the ^4^F_3/2_ level at 123 K (e); comparison of decay curves (f) and average decay times (g) of representative NaYF_4_ and NaGdF_4_ samples doped with 2% Nd^3+^ at 123 K and 298 K temperatures.

Trivalent neodymium ions, due to their emission and excitation electronic transitions occurring in the near infrared range (NIR), are one of the most favorable ions for luminescence thermometry dedicated for biological applications. Moreover, as can be seen in the schematic diagram of energy levels in Fig. 2a, they are characterized by a dense ladder of energy levels, whereby the energy difference between the ^4^I_9/2_ and ^4^I_11/2_ levels of the ground multiplet is in the order of 2000 cm^−1^, which can be overcome by the supplied thermal energy. Therefore, the excited state absorption process, necessary in single-band ratiometric luminescence thermometry, would become possible. Furthermore, as indicated by the yellow arrows in Fig. 2a, the energy levels exhibit an energy match that enables efficient (^4^F_3/2_; ^4^I_9/2_) → (^4^I_15/2_; ^4^I_15/2_) cross-relaxation (CR) to occur, the probability of which increases proportionally to the concentration of optically active ions. This process results in an increase in the ^4^I_15/2_ level population followed by nonradiative depopulation to lower ^4^I_*J*_ levels (grey arrows in Fig. 2a), indicating that CR may be one of the effective population channels of the ^4^I_11/2_ level population and thus may contribute to the increased probability of the ESA process involved in SBR LT. To optimize the GSA-excitation wavelength, the excitation spectra monitored at 1058 nm were recorded and analyzed for all the samples considered (Fig. 2b and S3a† for NaYF_4_:Nd^3+^ and NaGdF_4_:Nd^3+^, respectively). It reveals that although several excitation transitions are possible, the largest excitation cross-section is associated with a wavelength of 793 nm (indicated by the pink arrow in Fig. 2a), which allows an electronic transition from the ^4^I_9/2_ level to the ^4^F_5/2_, ^2^H_9/2_ level, from which an immediate nonradiative relaxation to the ^4^F_3/2_ level occurs. Moreover, the 793 nm line fits into the NIR range, which is very favorable for luminescence thermometry, so in this work this wavelength was chosen for GSA excitation. The emission spectra obtained at this excitation wavelength consist of three emission bands at 880 nm, 1058 nm, and 1335 nm, which are associated with the ^4^F_3/2_ → ^4^I_*J*_ transitions (*J* = 9/2, 11/2, and 13/2, respectively) (Fig. 2c, inset and Fig. S4†). Careful analysis of the emission spectra of different concentrations of Nd^3+^ ions in the NaYF_4_ host, normalized to the intensity of the 1058 nm band (Fig. 2c, complementary data for NaGdF_4_ can be seen in Fig. S3b†) reveals a significant decrease in the intensity and the change in the shape of the ^4^F_3/2_ → ^4^I_9/2_ emission band at 880 nm with increasing dopant ion concentration. The observed decrease of the intensity of lines localized at shorter wavelengths (corresponding to the electronic transition between R_1_ and R_2_ Stark sublevels of the ^4^F_3/2_ state to the Z_1_ Stark sublevel of the ^4^I_9/2_ state) with respect to the total emission band at elevated Nd^3+^ concentration results from the reabsorption process. Since the energy of the emitted light is in resonance with the absorption of the next neighboring Nd^3+^ ions, it can be easily reabsorbed modifying the shape of the emission band. It is worth noting that the emission intensity of the bands is strongly dependent on the concentration of Nd^3+^ ions. In both NaYF_4_ and NaGdF_4_ hosts, the intensity of all emission bands increases with increasing concentration from 0.1% to 2% Nd^3+^, which is attributed to the increasing absorption cross-section, *i.e.*, the larger the number of optically active ions capable of absorption, the larger the active cross-section. On the other hand, for concentrations of 5% and higher, a decrease in emission intensity becomes evident, which should be related to the rising role of the cross-relaxation, which, at the expense of populating the ^4^F_3/2_ level, increases the occupancy of the ^4^I_15/2_ level followed by its nonradiative depopulation to lower ^4^I_*J*_ manifolds. This was confirmed by the shortening of the luminescence lifetime of the ^4^F_3/2_ state with increasing dopant ion concentration (Fig. 2d and S3c† for Nd^3+^-doped NaYF_4_ and NaGdF_4_, respectively). The average luminescence lifetimes presented in Fig. 2e (and Fig. S3d† for NaGdF_4_:Nd^3+^ nanocrystals) were calculated using the following equation:1
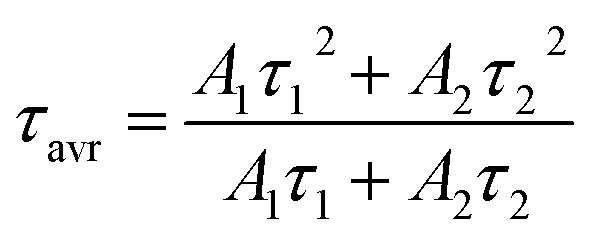
where all the parameters were obtained from the fitting of the experimental decay profile with a double exponential function (eqn (S1)†); *A*_*i*_ (*i* = 1 and 2) are the amplitudes of the respective components, *τ*_*i*_ are the individual lifetimes determined as *τ*_*i*_ = *t*_*i*_ × ln(2), where *t*_*i*_ means the time constant (see Fig. S5†). With an increase of Nd^3+^ ion concentration from 0.1% to 50%, a gradual shortening of the average luminescence lifetime from 165 μs to 8.2 μs and from 196 μs to 8.4 μs in NaYF_4_:Nd^3+^ and NaGdF_4_:Nd^3+^, respectively, is evident, which confirms CR related concentration quenching.

To investigate the sensitivity of the decay times to temperature changes, the kinetics of the excited ^4^F_3/2_ state at 123 K and 298 K in representative samples of both hosts doped with 2% Nd^3+^ ions were compared (Fig. 2f). In general, luminescence decay times were observed to be slightly longer in the NaGdF_4_ host; however, gently greater sensitivity to temperature changes was observed in the NaYF_4_ host. As shown in Fig. 2g, when the temperature was increased from 123 K to 298 K, the lifetime of Nd^3+^ luminescence decreased by 0.019 ms (from 0.109 ms to 0.090 ms) in the NaYF_4_ host, while in NaGdF_4_ it decreased by 0.015 ms (from 0.116 ms to 0.101 ms).

In addition to the above-described process of photon generation occurring on the GSA path, under certain conditions, *i.e.*, when the ^4^I_11/2_ level is sufficiently populated, the ESA process using an excitation wavelength of 1060 nm can also occur (turquoise arrow in the energy diagram in Fig. 2a). Several mechanisms may contribute to the population of this level such as: (1) *via* (^4^F_3/2_; ^4^I_9/2_) → (^4^I_15/2_; ^4^I_15/2_) cross-relaxation (yellow arrows in Fig. 2a), followed by non-radiative transition to the ^4^I_11/2_ state (grayish wavy arrows in Fig. 2a); (2) as a result of ^4^F_3/2_ → ^4^I_11/2_ emission transitions, which are characterized by a nearly 60% branching ratio or (3) by thermalization of the excited level with electrons from the ground state consistent with Boltzmann distribution. The latter mechanism is key for luminescence thermometry with ESA based luminescence.

**Fig. 3:**
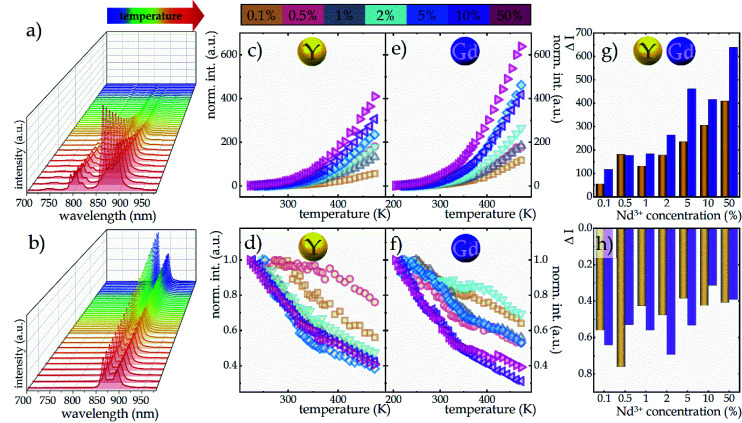
Representative thermal evolution of the ^4^F_3/2_ → ^4^I_9/2_ emission band at 880 nm upon 1060 nm (a) and 793 nm (b) excitation; the influence of the dopant concentration on the thermal evolution of normalized integral emission intensities of NaYF_4_ and NaGdF_4_ nanocrystals upon ESA (c and e, respectively) and GSA (d and f, respectively) excitation; the degree of change in the intensity of ESA (g) and GSA (h) excited luminescence of Nd^3+^ ions in NaYF_4_ and NaGdF_4_ as a function of dopant ion concentration determined over a temperature range: from the minimum temperature at which ESA excitation occurred to a temperature of 473 K.

In order to visualize the influence of temperature change on the emission intensity of Nd^3+^ ions, the thermal evolution of the emission spectra of representative NaYF_4_:2%Nd^3+^ nanocrystals limited to the emission band at 880 nm associated with the ^4^F_3/2_ → ^4^I_9/2_ electronic transition (bold purple arrow in Fig. 2a) under both ESA and GSA excitation conditions are presented in Fig. 3a and b, respectively. As expected, in the case of ESA excitation at low temperatures when the population of the ^4^I_11/2_ level is negligible, the emission band is not observed; however, when the thermalization of this level occurs at increased temperatures, a sharp increase in intensity is noted. Moreover, as can be seen at higher temperatures, in addition to the analyzed band at 880 nm, two other emission bands at 800 nm (above 328 K) and 750 nm (above 413 K) associated with the ^4^F_5/2_, ^2^H_9/2_ → ^4^I_9/2_ and ^4^F_7/2_, ^4^S_3/2_ → ^4^I_9/2_ electronic transitions, respectively (indicated in Fig. 2a by thin purple arrows) are also visible. This effect is due to the thermalization of higher ^4^F_5/2_, ^2^H_9/2_ and ^4^F_7/2_, ^4^S_3/2_ levels above metastable ^4^F_3/2_. This is feasible because the energy differences between both ^4^F_3/2_ and ^4^F_5/2_, ^2^H_9/2_ levels and between ^4^F_5/2_, ^2^H_9/2_ and ^4^F_7/2_, ^4^S_3/2_ levels equal *ca.* 1000 cm^−1^. On the other hand, the opposite thermal dependence, *i.e.*, the decrease in intensity, of the same ^4^F_3/2_ → ^4^I_9/2_ emission band being excited by GSA is evident (Fig. 3b). For qualitative comparison, the effect of dopant ion concentration on the integral emission intensities of NaYF_4_:Nd^3+^ and NaGdF_4_:Nd^3+^ nanocrystals upon ESA excitation is shown in Fig. 3c and e, respectively. As can be seen, an improvement in ESA process efficiency with increasing temperature resulting in a strong increase in emission intensity was noted; however, the rate of increase was strongly dependent on the dopant ion concentration and the host. From the comparison of the intensity changes Δ*I* = *I*_max_/*I*_0_ (Fig. 3g and h), where *I*_max_ is the intensity at the maximum analyzed temperature (473 K) and *I*_0_ is the initial intensity, at the temperature where the ESA excitation-induced emission appears, it is clearly seen that increasing the Nd^3+^ concentration from 0.1% to 50% resulted in a gradual enhancement in the growth rate. This in consequence leads to more than 7-fold in the NaYF_4_ and more than 5-fold in the NaGdF_4_ host thermally induced changes in the intensity of ESA-excited luminescence. The gradual increase in the intensity growth rate for nanocrystals with a higher Nd^3+^ concentration, indicating the occurrence of the CR phenomenon, is a confirmation that increased doping with optically active ions has a beneficial effect on ESA-excited luminescence. On the other hand, CR also affects the depopulation of the ^4^F_3/2_ emitting level, as captured in the integral intensities of NaYF_4_:Nd^3+^ and NaGdF_4_:Nd^3+^ nanocrystals upon GSA excitation presented in Fig. 3d and f. In general, an increase in temperature induces multiphonon relaxation contributing to a decrease in emission intensity; however, the probability of this process is independent of the concentration of the optically active ions. Therefore, the thermally activated CR process is responsible for the dependence of intensity changes with increasing temperature on dopant ion concentration. In general the probability of CR is temperature independent. However, when some energy mismatch occurs the CR is activated with the assistance of phonons, leading to its strong thermal susceptibility. At a concentration of 0.1%, a decrease in Δ*I* intensity of about 45% and about 35% relative to the initial value in the NaYF_4_ and NaGdF_4_ hosts, respectively, was observed, whereas increasing the concentration of Nd^3+^ ions to 50% resulted in a much stronger quenching of about 60% independently of the host (see Fig. 3h). It should be noted that in the case of nanoscale phosphors, one of the important channels for the depopulation of excited levels is surface-related quenching processes, which can significantly affect the temperature dependence of the emission intensity of Nd^3+^ ions upon both GSA and ESA excitation.^32^ In the case of nanocrystals, the clustering of ions within the surface is often observed.^22^ As a result, an increase in the concentration of Nd^3+^ ions may result in not only an enhanced probability of CR processes, but also in an increased number of ions affected by surface processes. In order to verify this hypothesis, the homogeneity of the distribution of Nd^3+^ ions within the nanocrystal must be confirmed. For this purpose, the change in unit cell volume as a function of Nd^3+^ ion concentration was analyzed (Fig S6†). A linear correlation was observed suggesting a homogeneous ion distribution, which excludes a dominant role of surface effects.

The opposite monotonicity of thermal dependence of the 880 nm emission band upon ESA and GSA led to the development of a single-band ratiometric luminescence thermometer in which the temperature-dependent parameter is determined by the luminescence intensity ratio (LIR) defined as follows:2
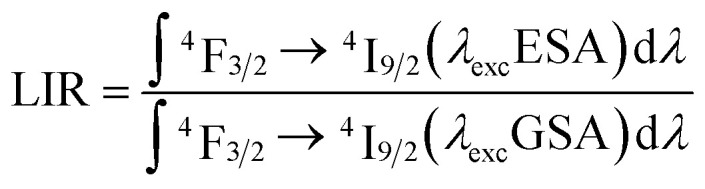


**Fig. 4:**
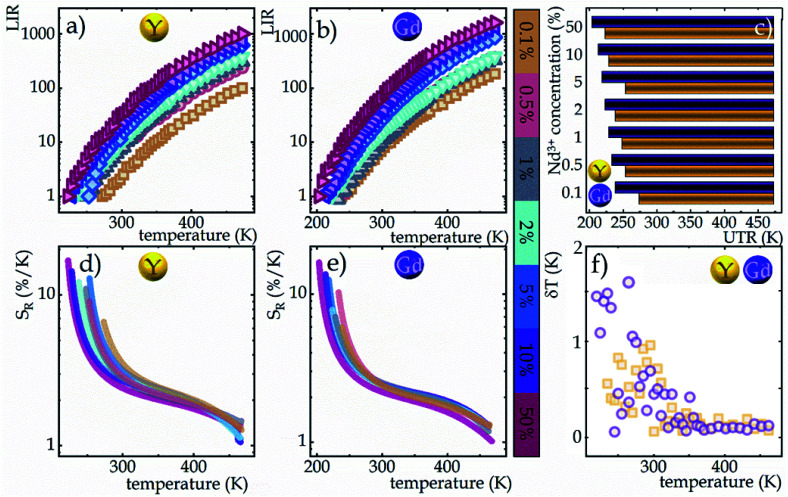
The temperature dependent LIR values of NaYF_4_ (a) and NaGdF_4_ (b) doped with Nd^3+^ ions in concentrations ranging from 0.1 to 50%; influence of dopant ion concentration on the useful temperature range (c); the relative sensitivities of Nd^3+^-doped NaYF_4_ and NaGdF_4_ based SBR LTs (d and e, respectively), and temperature resolution of representative SBR LTs in NaYF_4_ and NaGdF_4_ doped with 2% Nd^3+^ ions (f).

Due to the greater susceptibility of ESA-excited luminescence to temperature changes than the GSA-excited one, the trend of changes in the LIR values is governed mostly by the changes observed in ESA-excited luminescence. Regardless of the concentration of Nd^3+^ ions and the host type, the LIR values increase over the entire temperature range analyzed; however, the rate of these changes is dependent on the dopant ion concentration and material analyzed (Fig. 4a and b). For the lowest analyzed concentration of 0.1% Nd^3+^ ions, after increasing the temperature to 473 K, a 100-fold and 180-fold increase in the LIR values in the NaYF_4_ and NaGdF_4_ host, respectively, relative to the initial value was evident. Nevertheless, increasing the concentration to 50% Nd^3+^ ions allowed an almost 10-fold improvement in the changes of LIR under the same temperature conditions, achieving spectacular 1000-fold and 1600-fold enhancement of the LIR value relative to its initial value. Furthermore, it is worth noting here that due to the fact that the increased concentration, as a result of CR, is an additional effective channel of the population of the ^4^I_11/2_ level, the higher the dopant ion concentration the greater the population density at this level. Due to the high population density of this level, the ESA-excited luminescence of Nd^3+^ ions is visible also at low temperatures, which contributes to a gradual expansion of the useful temperature range (UTR) of a given thermometer (Fig. 4c). Changing the concentration from 0.1% Nd^3+^ to 50% Nd^3+^ results in a widening of the UTR from 200 K (operating range 273 K to 473 K) to 250 K (223–473 K) in the NaYF_4_ host and from 235 K (238–473 K) to 270 K (203–473 K) in the NaGdF_4_ nanocrystals.

In order to quantify the ability of these SBR-based LTs to perform temperature readout, the relative sensitivities to thermal changes were determined according to the following equation:3
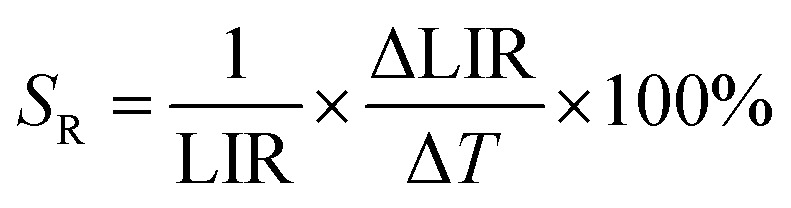
where ΔLIR represents the change of the LIR value caused by changes in temperature Δ*T*. The calculated relative sensitivities of NaYF_4_:Nd^3+^ and NaGdF_4_:Nd^3+^ nanocrystals are shown in Fig. 4d and e, respectively. In the temperature range from 200 K to 300 K, a gradual increase in relative sensitivity and a widening of the useful temperature range with increasing concentration were noted. As expected, regardless of the host, the highest sensitivities were obtained for materials containing 50% Nd^3+^ ions and reached 16.9% K^−1^ at 223 K for NaYF_4_:50%Nd^3+^ and 16.3% K^−1^ at 203 K for NaGdF_4_:50%Nd^3+^, respectively. As the temperature increased, their value decreased; however, regardless of the concentration of Nd^3+^ ions and the host type, it did not drop below 1% K^−1^ over the entire temperature range analyzed. Motivated by such unprecedentedly high relative sensitivities to confirm the ability of the analyzed nanocrystals to determine temperature, the temperature resolutions were determined according to the following formula:4
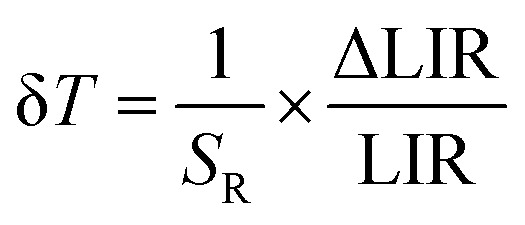


The temperature resolutions of representative SBR LTs in NaYF_4_ and NaGdF_4_ nanocrystals doped with 2% Nd^3+^ ions are presented in Fig. 4f. In the temperature range of 200–300 K, the average δ*T* values deviated from the desired low values and reached up to 5 K depending on the host type (Fig. 4f) and dopant ion concentration (see Fig. S7†). However, once those temperatures were exceeded, excellent average values of temperature resolution of about 0.1–0.2 K were observed for optimal 2% Nd^3+^ ions regardless of the host type. For the other concentrations (Fig. S7†), the values do not satisfy the condition of temperature resolution <0.1 K ^33^ and are >0.5 K. The reason for the weakened temperature resolution in the low temperature range is probably the relatively large error derived from the small signal-to-noise ratio present in this temperature range for ESA-induced luminescence. Therefore, despite the very high and very promising relative sensitivities of the analyzed luminescence thermometers, the reliability of the temperature readout in the low temperature range may be insufficient for practical applications. Nevertheless, the single-band ratiometric luminescent thermometers presented in this work, particularly those based on materials doped with 2% Nd^3+^, exhibit outstanding thermometric properties over a wide temperature range from 300 to 473 K. Although it would seem that due to the increasing brightness (resulting from the increased number of emitting ions) and the increase in ESA a special attention should be paid to materials doped with a high concentration of optically active ions, some balance must be found with concentration quenching processes (resulting from the increase in cross relaxation). Moreover, it is important to highlight the fact that the heavily Nd^3+^ doped NaYF_4_ nanoparticles exhibit efficient light-to-heat conversion,^34,35^ which may strongly disturb accurate temperature readout. Due to all the phenomena mentioned above, we consider NaYF_4_:2%Nd^3+^ and NaGdF_4_:2%Nd^3+^ nanocrystals to be optimal for single-band ratiometric luminescence thermometry applications.

## Conclusions

In the present work, two series of NaYF_4_:Nd^3+^ and NaGdF_4_:Nd^3+^ fluoride nanocrystals with the Nd^3+^ ion concentrations ranging from 0.1 to 50% and with sizes below 20 nm were synthesized by thermal decomposition. The effects of dopant ion concentration and material parameters on spectroscopic and thermometric properties over a wide temperature range from 200 to 473 K have been investigated. Thermometric studies were performed using two excitation wavelengths matching absorption from the ground state (*λ*_EXC_ = 793 nm) and from the excited state (*λ*_EXC_ = 1060 nm) in order to investigate the suitability of the analyzed materials for single-band ratiometric luminescence thermometry. The wavelength of 793 nm was chosen, among other reasons, because of the high absorption cross-section of NaYF_4_:Nd^3+^ and NaGdF_4_:Nd^3+^ nanocrystals in this spectral range and due to its occurrence in the near-infrared range. The latter feature makes them suitable for biological applications. To determine the temperature, the emission intensity of the band at 880 nm associated with the ^4^F_3/2_ → ^4^I_9/2_ electronic transition under the aforementioned excitation conditions was used. Owing to the fact that two other physical processes determine the nature of the thermally induced emission intensity changes depending on which excitation wavelength is involved, *i.e.*, the thermal population of the ^4^I_11/2_ level in the case of ESA excitation and the nonradiative multiphoton depopulation of the ^4^F_3/2_ level determining the temperature evolution of the emission spectra upon GSA excitation, two distinct trends are observed, *i.e.*, a strong increase in the ESA-excited emission intensity with increasing temperature and a decrease in the GSA-excited emission intensity. The determined temperature-dependent LIR parameter revealed very pronounced changes with increasing temperature, with the rate of changes being strongly dependent on the concentration of Nd^3+^ ions. After increasing the temperature from the initial to 473 K, about 100- and 180-fold enhancement in the LIR value was noted for the NaYF_4_ and NaGdF_4_ hosts doped at the lowest degree of 0.1% Nd^3+^. However, a nearly 10-fold improvement over these values of LIR was obtained when the concentration was increased up to 50% Nd^3+^, which overall demonstrated a spectacular 1000-fold (NaYF_4_:50%Nd^3+^) increase and 1600-fold (NaGdF_4_: 50%Nd^3+^) enhancement of the LIR value relative to its initial value. This several-fold improvement in thermally induced changes in LIR values with increasing concentration confirms the beneficial effect of cross-relaxation on luminescence intensity, which is not commonly observed. Moreover, increasing the concentration of Nd^3+^ ions, due to the efficient population of the ^4^I_11/2_ level through cross-relaxation, contributed to an increased electron density at this level, resulting in the capability to observe the ESA-excited luminescence even at low temperatures and thus expanding the useful temperature range. In particular, changing the concentration from 0.1% Nd^3+^ to 50% Nd^3+^ widened the UTR from 200 K (operating range 273 K to 473 K) to 250 K (223–473 K) in the NaYF_4_ host and from 235 K (238–473 K) to 270 K (203–473 K) in the NaGdF_4_ nanocrystals. Of all the materials analyzed, the highest relative sensitivities were obtained in materials doped with 50% Nd^3+^ ions, regardless of the host, and they reached 16.9% K^−1^ at 223 K for NaYF_4_:50%Nd^3+^ and 16.3% K^−1^ at 203 K for NaGdF_4_:50%Nd^3+^, respectively. As the temperature increased, their value decreased; however, regardless of the concentration of Nd^3+^ ions and the host type, it was kept above the critical 1% K^−1^ value over the entire temperature range analyzed. These unprecedentedly high values indicate the excellent potential of the analyzed nanocrystals for luminescence thermometry applications, which was confirmed by the determination of the temperature resolutions, the values of which were about 0.1–0.2 K in the temperature range of 300–473 K for the optimal 2% Nd^3+^ concentration regardless of the host analyzed.

## Conflicts of interest

There are no conflicts to declare.

## Supplementary Material

NA-004-D1NA00727K-s001
