# Correction: A single-band ratiometric luminescent thermometer based on tetrafluorides operating entirely in the infrared region

**DOI:** 10.1039/d2na90035a

**Published:** 2022-05-12

**Authors:** K. Trejgis, K. Ledwa, A. Bednarkiewicz, L. Marciniak

**Affiliations:** Institute of Low Temperature and Structure Research, Polish Academy of Sciences Okolna 2 50-422 Wroclaw Poland k.trejgis@intibs.pl l.marciniak@intibs.pl

## Abstract

Correction for ‘A single-band ratiometric luminescent thermometer based on tetrafluorides operating entirely in the infrared region’ by K. Trejgis *et al.*, *Nanoscale Adv.*, 2022, **4**, 437–446, https://doi.org/10.1039/D1NA00727K.

The authors regret the omission of a funding acknowledgement in the original article. This acknowledgement is given below.

Karolina Trejgis would like to acknowledge support from the Foundation for Polish Science (FNP).

The Royal Society of Chemistry apologises for these errors and any consequent inconvenience to authors and readers.

## Supplementary Material

