# Supplementary material for: A single-band ratiometric luminescent thermometer based on tetrafluorides operating entirely in the infrared region
Source: Nanoscale Adv. 2021 Nov 12;4(2):437–46. doi: 10.1039/d1na00727k (PMC9416883; doi:10.1039/d1na00727k)
Supplement: NA-004-D1NA00727K-s001 [file NA-004-D1NA00727K-s001.pdf]

Electronic Supplementary Material (ESI) for Nanoscale Advances.
This journal is © The Royal Society of Chemistry 2021

Supporting Information

A single-band ratiometric luminescent thermometer based on tetrafluorides operating entirely in the infrared region

K. Trejgis[1*], K. Ledwa[1], A. Bednarkiewicz[1], L. Marciniak[1*]

[1]Institute of Low Temperature and Structure Research, Polish Academy of Sciences, Okolna 2, 50-422 Wroclaw, Poland

* corresponding author: k.trejgis@intibs.pl, *l.marciniak@intibs.pl*

KEYWORDS excited state absorption, SBR approach, luminescence thermometry, fluoride, neodymium

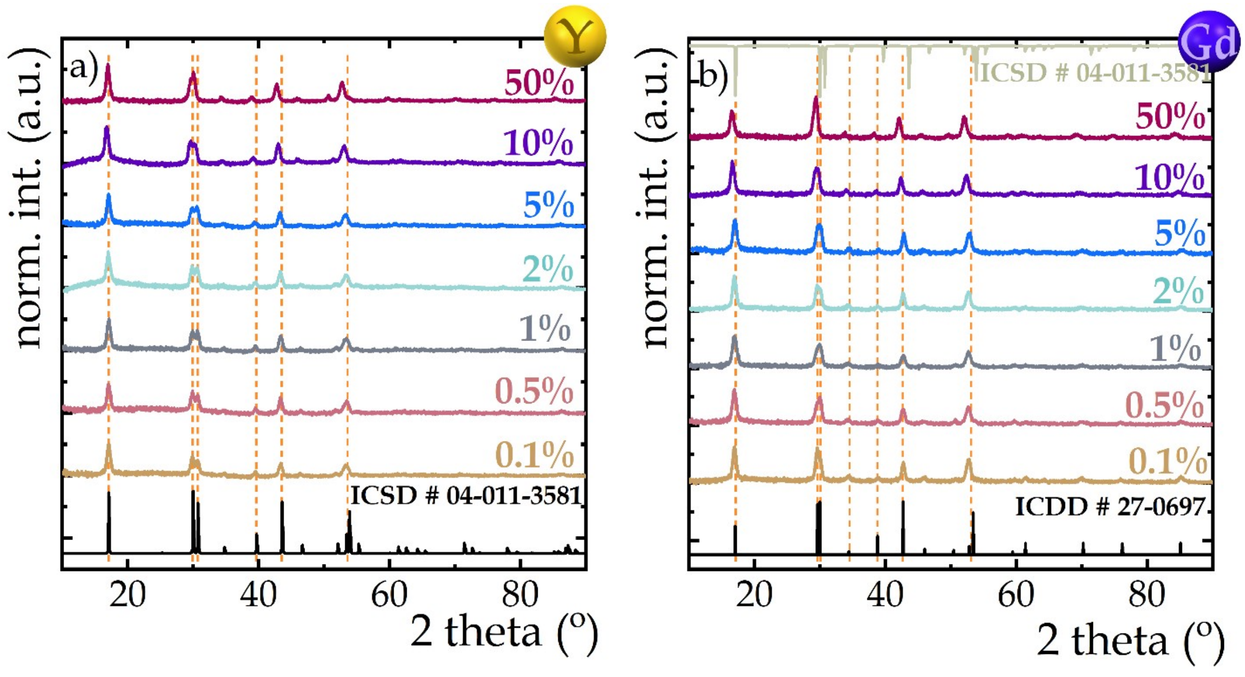

Figure S1. Shift of Bragg reflections towards lower angles for high concentrations of Nd^{3+} ions in $NaYF_4$ (a) and $NaGdF_4$ (b) host. At the top of figure b) a pattern for $NaYF_4$ is also shown,

allowing comparison of the shift of Bragg reflections in the $NaGdF_4$ matrix relative to those in the $NaYF_4$ counterpart.

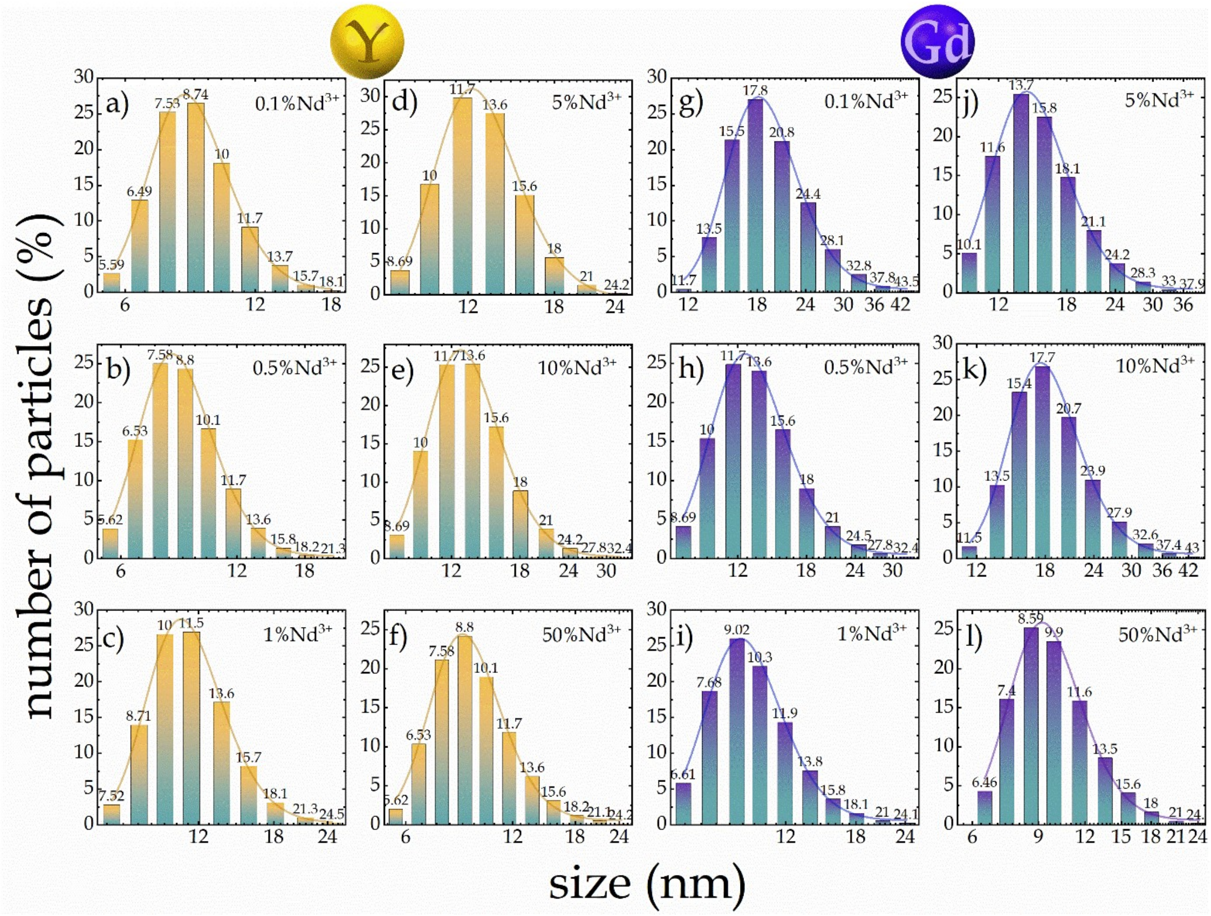

Figure S2. The hydrodynamic size distribution of representative $NaYF_4$ (a-f) and $NaGdF_4$: (g-l) doped with different Nd^{3+} concentration ranging from 0.1% to 50% determined from DLS measurement.

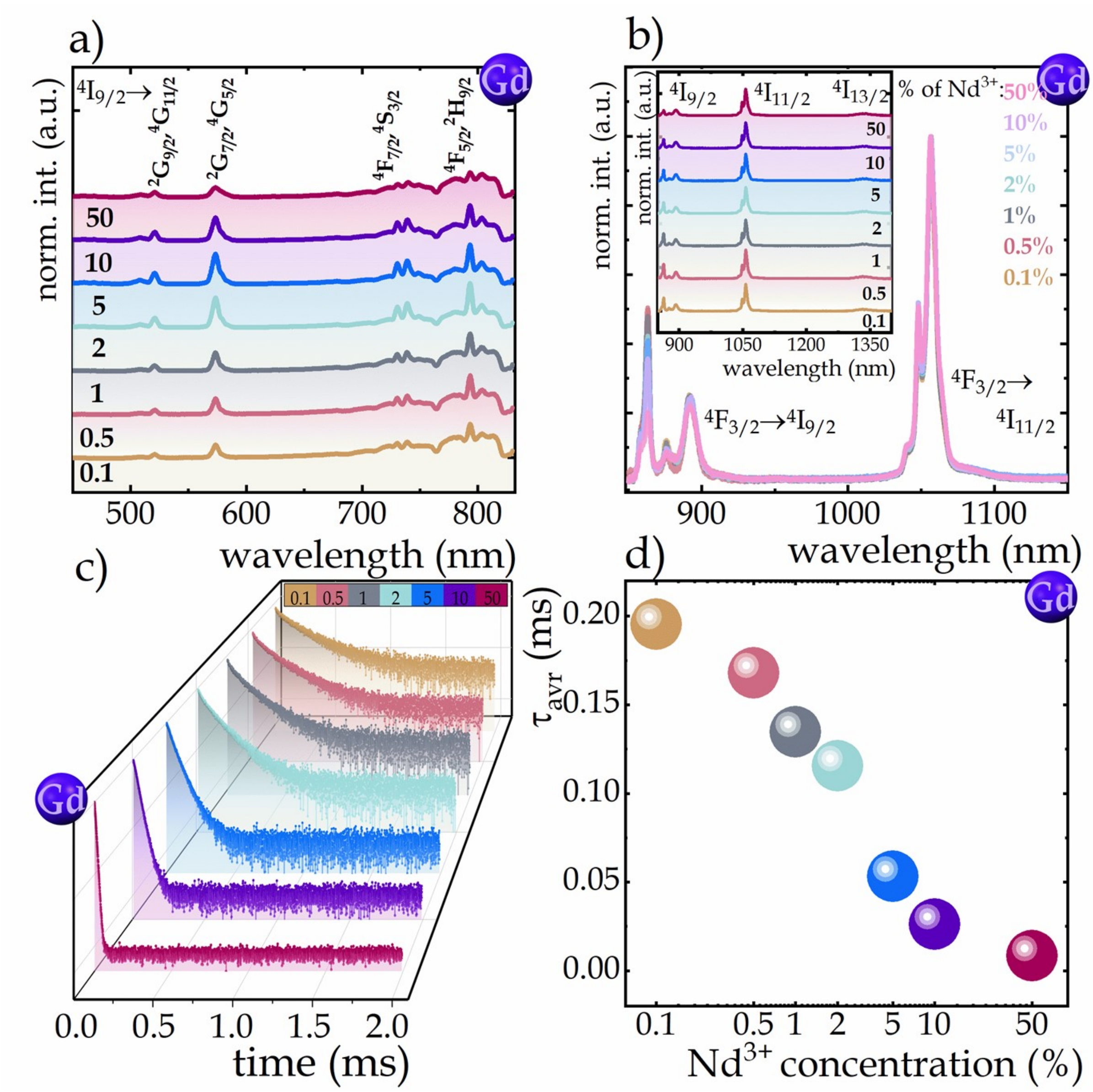

Figure S3. Nd^{3+} ions concentration impact on excitation spectra monitored at 1058 nm (a), emission spectra upon 793 nm excitation (b), the kinetics at 123 K of the excited $^4F_{3/2}$ state (c) and average decay time of $^4F_{3/2}$ level at 123K (d) in $NaGdF_4$ host.

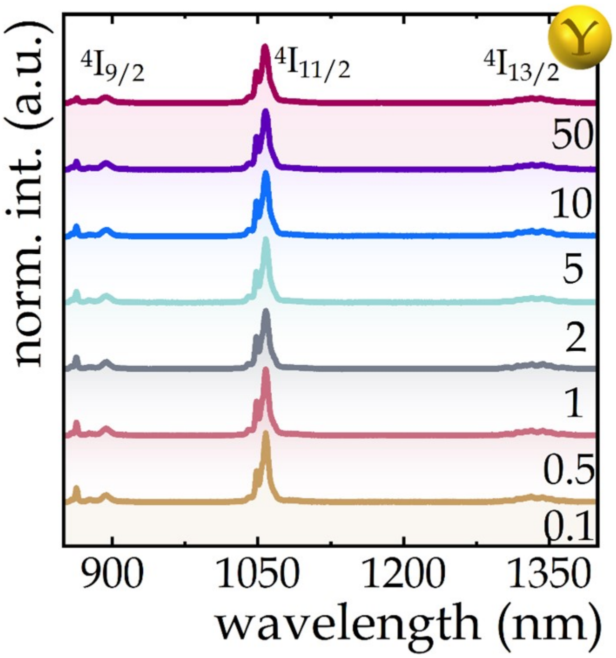

Figure S4. The comparison of the emission spectra upon 793 nm excitation for $NaYF_4$:Nd^{3+}.

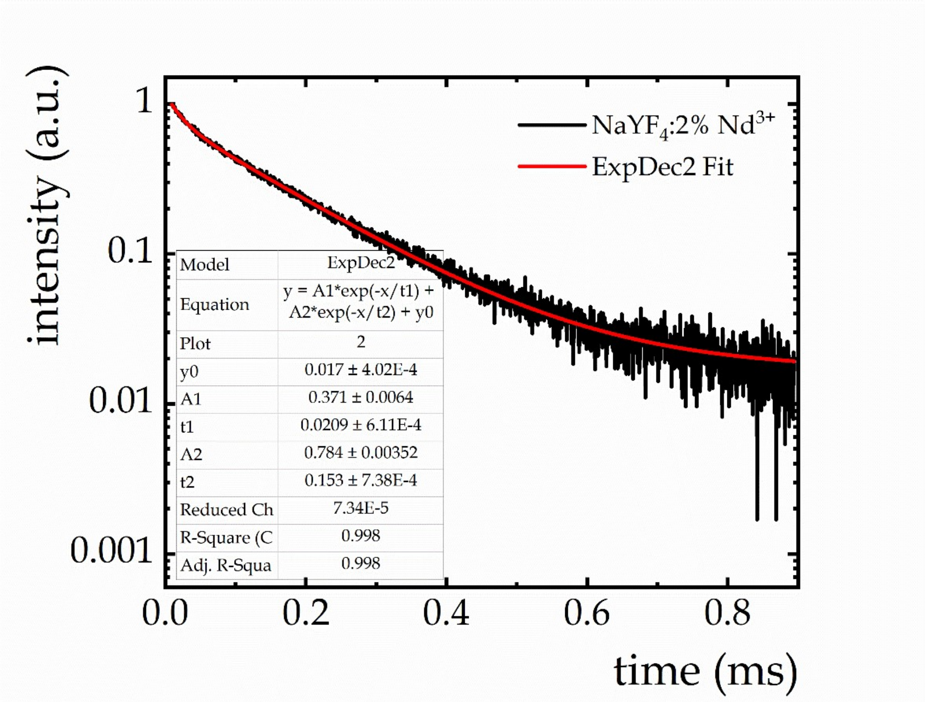

Figure S5. Decay curve and fit curve of the ExpDec2 function of an example $NaYF_4$:2%Nd^{3+} sample. The table shows the parameter values used to calculate the average time with the double-exponential function according to Equation 1.

Equation in the two-exponential ExpDec2 model:

$$y = A_1 \cdot e^{-x/t_1} + A_2 \cdot e^{-x/t_2} + y_0 \qquad \text{(eq. S1)}$$

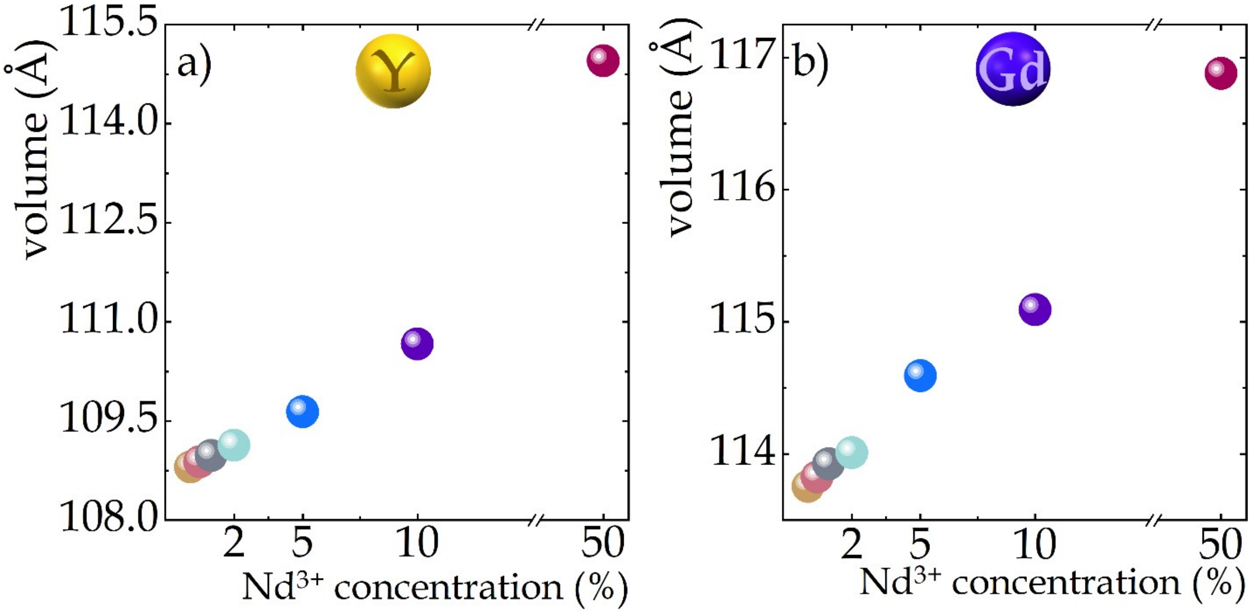

Figure S6. Dependence of unit cell volume on the concentration of Nd^{3+} ions in the host $NaYF_4$ (a) and $NaGdF_4$ (b)

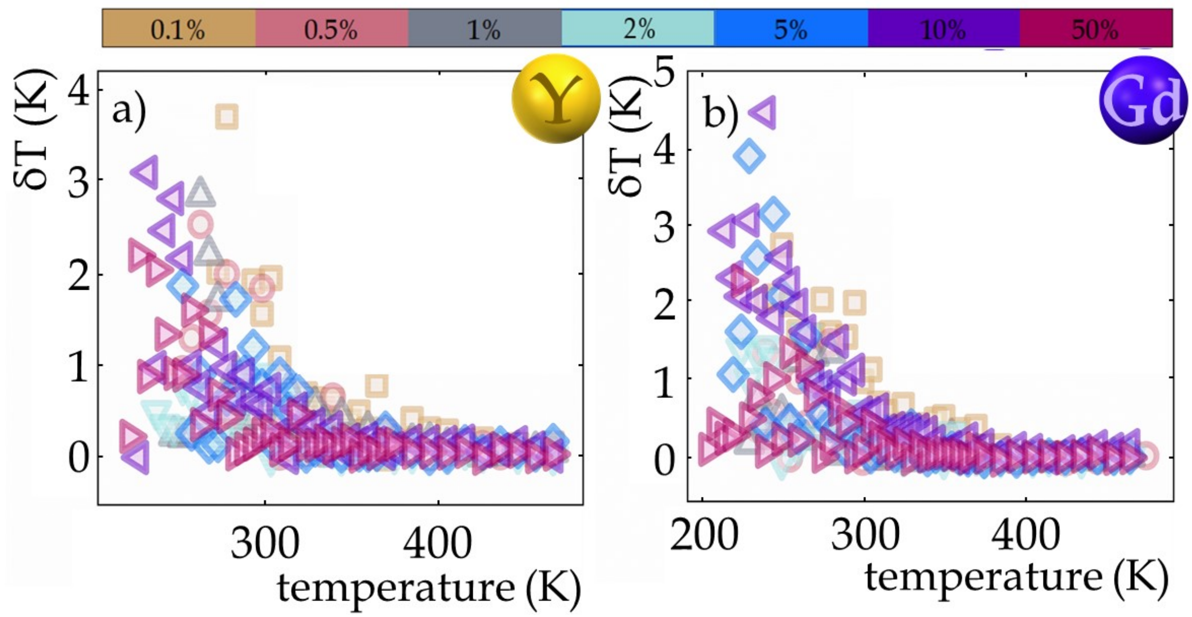

Figure S7. The temperature resolution of Nd^{3+}-doped $NaYF_4$ and $NaGdF_4$ based LTs (a and b, respectively).
